# Supplementary material for: Transcriptomic Analysis of Liver Tissue of Black Sea Bass (Centropristis striata) Exposed to High Nitrogen Environment
Source: Genes (Basel). 2023 Jul 13;14(7):1440. doi: 10.3390/genes14071440 (PMC10378819; doi:10.3390/genes14071440)
Supplement: Supplementary file 1 [file genes-14-01440-s001.zip › Figure S2.pdf]

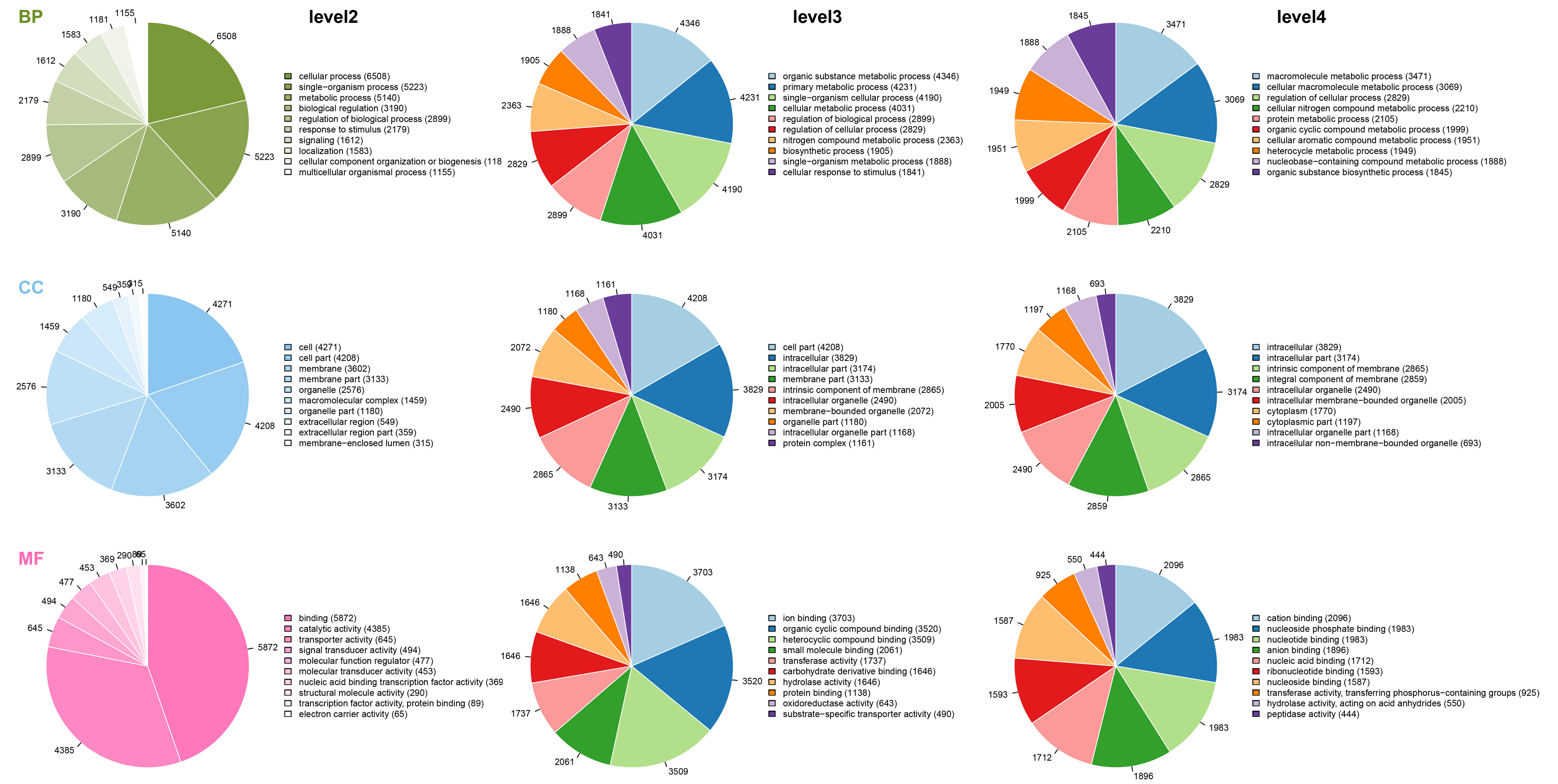
BP
level2
cellular process (6508)
single−organism process (5223)
metabolic process (5140)
biological regulation (3190)
regulation of biological process (2899)
response to stimulus (2179)
signaling (1612)
localization (1583)
cellular component organization or biogenesis (118
multicellular organismal process (1155)
level3
organic substance metabolic process (4346)
primary metabolic process (4231)
single−organism cellular process (4190)
cellular metabolic process (4031)
regulation of biological process (2899)
regulation of cellular process (2829)
nitrogen compound metabolic process (2363)
biosynthetic process (1905)
single−organism metabolic process (1888)
cellular response to stimulus (1841)
level4
macromolecule metabolic process (3471)
cellular macromolecule metabolic process (3069)
regulation of cellular process (2829)
cellular nitrogen compound metabolic process (2210)
protein metabolic process (2105)
organic cyclic compound metabolic process (1999)
cellular aromatic compound metabolic process (1951)
heterocycle metabolic process (1949)
nucleobase−containing compound metabolic process (1888)
organic substance biosynthetic process (1845)
CC
cell (4271)
cell part (4208)
membrane (3602)
membrane part (3133)
organelle (2576)
macromolecular complex (1459)
organelle part (1180)
extracellular region (549)
extracellular region part (359)
membrane−enclosed lumen (315)
cell part (4208)
intracellular (3829)
intracellular part (3174)
membrane part (3133)
intrinsic component of membrane (2865)
intracellular organelle (2490)
membrane−bounded organelle (2072)
organelle part (1180)
intracellular organelle part (1168)
protein complex (1161)
intracellular (3829)
intracellular part (3174)
intrinsic component of membrane (2865)
integral component of membrane (2859)
intracellular organelle (2490)
intracellular membrane−bounded organelle (2005)
cytoplasm (1770)
cytoplasmic part (1197)
intracellular organelle part (1168)
intracellular non−membrane−bounded organelle (693)
MF
binding (5872)
catalytic activity (4385)
transporter activity (645)
signal transducer activity (494)
molecular function regulator (477)
molecular transducer activity (453)
nucleic acid binding transcription factor activity (369
structural molecule activity (290)
transcription factor activity, protein binding (89)
electron carrier activity (65)
ion binding (3703)
organic cyclic compound binding (3520)
heterocyclic compound binding (3509)
small molecule binding (2061)
transferase activity (1737)
carbohydrate derivative binding (1646)
hydrolase activity (1646)
protein binding (1138)
oxidoreductase activity (643)
substrate−specific transporter activity (490)
cation binding (2096)
nucleoside phosphate binding (1983)
nucleotide binding (1983)
anion binding (1896)
nucleic acid binding (1712)
ribonucleotide binding (1593)
nucleoside binding (1587)
transferase activity, transferring phosphorus−containing groups (925)
hydrolase activity, acting on acid anhydrides (550)
peptidase activity (444)
