# Supplementary material for: Transcriptomic Analysis of Liver Tissue of Black Sea Bass (Centropristis striata) Exposed to High Nitrogen Environment
Source: Genes (Basel). 2023 Jul 13;14(7):1440. doi: 10.3390/genes14071440 (PMC10378819; doi:10.3390/genes14071440)
Supplement: Supplementary file 1 [file genes-14-01440-s001.zip › Table S1.pdf]

Table S1. Summary of RNA-Seq de novo assembly results

Assembly	Info
Total unigenes num:	84,603
Total unigenes length:	73,791,352
Total isoform num:	137,862
Total isoform length:	147,461,435
Average length:	872
Largest Unigene:	17,156
N50:	1,060
