# Supplementary material for: Transcriptomic Analysis of Liver Tissue of Black Sea Bass (Centropristis striata) Exposed to High Nitrogen Environment
Source: Genes (Basel). 2023 Jul 13;14(7):1440. doi: 10.3390/genes14071440 (PMC10378819; doi:10.3390/genes14071440)
Supplement: Supplementary file 1 [file genes-14-01440-s001.zip › Table S2.pdf]

Table S2. Control (ZD) vs. salt-treated (ZS) GO enrichment.

ID	Terms	Ratio in Study*	Ratio in Total Genes	p-value	Categories
GO:0010884	positive regulation of lipid storage	0.13%	0.01%	3.84E-12	biological process
GO:1905954	positive regulation of lipid localization	0.15%	0.02%	5.39E-12	biological process
GO:0010883	regulation of lipid storage	0.14%	0.02%	5.39E-12	biological process
GO:0050818	regulation of coagulation	0.18%	0.03%	1.06E-11	biological process
GO:1903034	regulation of response to wounding	0.18%	0.03%	1.06E-11	biological process
GO:0046890	regulation of lipid biosynthetic process	0.18%	0.03%	1.06E-11	biological process
GO:1900046	regulation of hemostasis	0.18%	0.03%	2.30E-11	biological process
GO:0030193	regulation of blood coagulation	0.18%	0.03%	2.30E-11	biological process
GO:0061041	regulation of wound healing	0.18%	0.03%	2.30E-11	biological process
GO:1900047	negative regulation of hemostasis	0.15%	0.02%	2.35E-11	biological process
GO:0030195	negative regulation of blood coagulation	0.39%	0.06%	2.35E-11	biological process
GO:0061045	negative regulation of wound healing	0.14%	0.02%	2.35E-11	biological process
GO:0051050	positive regulation of transport	0.14%	0.02%	2.45E-11	biological process
GO:0050878	regulation of body fluid levels	0.13%	0.02%	2.59E-11	biological process
GO:0072562	blood microparticle	0.16%	0.02%	3.11E-11	cellular component
GO:0033344	cholesterol efflux	0.95%	0.19%	3.43E-11	biological process
GO:0080134	regulation of response to stress	0.95%	0.19%	3.56E-11	biological process
GO:0034385	triglyceride-rich plasma lipoprotein particle	0.95%	0.19%	4.48E-11	cellular component
GO:0034361	very-low-density lipoprotein particle	1.03%	0.25%	4.48E-11	cellular component
GO:0042627	chylomicron	0.17%	0.02%	4.48E-11	cellular component
GO:0032269	negative regulation of cellular protein metabolic process	0.59%	0.19%	4.58E-11	biological process
GO:0052547	regulation of peptidase activity	0.60%	0.17%	4.71E-11	biological process
GO:0042613	MHC class II protein complex	0.52%	0.15%	4.78E-11	cellular component
GO:1905952	regulation of lipid localization	0.55%	0.16%	4.78E-11	biological process
GO:0052548	regulation of endopeptidase activity	0.52%	0.15%	4.80E-11	biological process
GO:0045861	negative regulation of proteolysis	1.01%	0.46%	4.80E-11	biological process
GO:0010033	response to organic substance	1.47%	0.77%	5.12E-11	biological process
GO:0030162	regulation of proteolysis	0.17%	0.03%	5.16E-11	biological process
GO:0070062	extracellular exosome	6.70%	5.19%	5.67E-11	cellular component
GO:0004857	enzyme inhibitor activity	0.61%	0.27%	5.67E-11	molecular function

* Ratio in study: Number of differentially expressed genes/total differentially expressed genes; Ratio in Total genes: Number of differential expressed genes/total genes.
