# Supplementary material for: Transcriptomic Analysis of Liver Tissue of Black Sea Bass (Centropristis striata) Exposed to High Nitrogen Environment
Source: Genes (Basel). 2023 Jul 13;14(7):1440. doi: 10.3390/genes14071440 (PMC10378819; doi:10.3390/genes14071440)
Supplement: Supplementary file 1 [file genes-14-01440-s001.zip › Table S3.pdf]

Table S3. Control (ZD) vs. salt-treated (ZS) Kyoto Encyclopedia of Genes and Genomes pathway enrichment.

Terms	Input number	Background number	P-Value	ID
Photosynthesis	31	32	3.54E-13	ko00195
Complement and coagulation cascades	71	264	8.20E-07	ko04610
Staphylococcus aureus infection	54	180	1.11E-06	ko05150
Asthma	19	36	9.90E-06	ko05310
Antigen processing and presentation	39	123	1.13E-05	ko04612
Photosynthesis - antenna proteins	10	10	3.26E-05	ko00196
Systemic lupus erythematosus	54	210	4.42E-05	ko05322
Intestinal immune network for IgA production	21	51	6.33E-05	ko04672
Graft-versus-host disease	24	72	0.000280729	ko05332
Autoimmune thyroid disease	24	72	0.000280729	ko05320
Oxidative phosphorylation	48	196	0.000295145	ko00190
Fatty acid degradation	32	113	0.000366486	ko00071
Terpenoid backbone biosynthesis	16	38	0.000375019	ko00900
Allograft rejection	24	74	0.000390246	ko05330
Type I diabetes mellitus	27	94	0.000845631	ko04940
Peroxisome	39	159	0.001001795	ko04146
Steroid biosynthesis	13	31	0.001333593	ko00100
Leishmaniasis	42	179	0.00137482	ko05140
Fat digestion and absorption	24	86	0.002191785	ko04975
Viral myocarditis	30	123	0.00383063	ko05416
Tryptophan metabolism	26	103	0.004646498	ko00380
Protein digestion and absorption	32	137	0.005023353	ko04974
Vitamin digestion and absorption	15	48	0.005990803	ko04977

Inflammatory bowel disease (IBD)	24	95	0.006302596	ko05321
Chloroalkane and chloroalkene degradation	13	40	0.007815983	ko00625
Parkinson's disease	46	227	0.009133606	ko05012
Glycolysis / Gluconeogenesis	37	175	0.010494004	ko00010
Rheumatoid arthritis	25	106	0.010950025	ko05323
Neuroactive ligand-receptor interaction	50	255	0.011380661	ko04080
Pancreatic secretion	39	193	0.015873592	ko04972
Bile secretion	33	157	0.015998236	ko04976
Phagosome	63	343	0.016058226	ko04145
Limonene and pinene degradation	8	21	0.016877325	ko00903
Tyrosine metabolism	18	72	0.017831781	ko00350
Glycerolipid metabolism	32	154	0.019815597	ko00561
PI3K-Akt signaling pathway	100	603	0.031714189	ko04151
Primary immunodeficiency	11	40	0.034435545	ko05340
Glyoxylate and dicarboxylate metabolism	17	73	0.034724745	ko00630
Steroid hormone biosynthesis	19	85	0.036399129	ko00140
Arginine and proline metabolism	26	128	0.040452298	ko00330
Linoleic acid metabolism	14	58	0.04198863	ko00591
Arachidonic acid metabolism	19	89	0.050762051	ko00590
beta-Alanine metabolism	14	61	0.056480053	ko00410
Nitrogen metabolism	6	18	0.056959309	ko00910
PPAR signaling pathway	27	140	0.059014611	ko03320
Betalain biosynthesis	3	5	0.059055364	ko00965
Retinol metabolism	20	97	0.059165442	ko00830
Other types of O-glycan biosynthesis	10	39	0.059167314	ko00514

Phototransduction - fly	13	56	0.060564058	ko04745
Valine, leucine and isoleucine degradation	19	92	0.063887508	ko00280
Aminobenzoate degradation	4	10	0.074749241	ko00627
Pyruvate metabolism	20	101	0.078327265	ko00620
Metabolism of xenobiotics by cytochrome P450	19	96	0.084760442	ko00980
One carbon pool by folate	11	48	0.084906251	ko00670
Histidine metabolism	14	66	0.087537276	ko00340
Glycosphingolipid biosynthesis - lacto and neolacto series	7	26	0.088627505	ko00601
Carbon fixation in photosynthetic organisms	13	61	0.094858856	ko00710
Butanoate metabolism	9	39	0.10913732	ko00650
Thiamine metabolism	4	12	0.112528588	ko00730
alpha-Linolenic acid metabolism	8	34	0.117992924	ko00592
Morphine addiction	25	140	0.119475813	ko05032
Chemical carcinogenesis	19	102	0.123620301	ko05204
Glycine, serine and threonine metabolism	19	102	0.123620301	ko00260
Tuberculosis	64	406	0.134472835	ko05152
Ovarian steroidogenesis	21	117	0.139146391	ko04913
GABAergic synapse	23	131	0.146013203	ko04727
Ascorbate and aldarate metabolism	14	73	0.146157452	ko00053
Naphthalene degradation	5	19	0.147638321	ko00626
Aminoacyl-tRNA biosynthesis	15	80	0.152453826	ko00970
Insulin secretion	29	172	0.154541679	ko04911
Fatty acid metabolism	19	106	0.154570021	ko01212
RNA polymerase	9	43	0.157643734	ko03020
Biosynthesis of amino acids	28	166	0.158997964	ko01230

Citrate cycle (TCA cycle)	14	75	0.166063375	ko00020
Cell adhesion molecules (CAMs)	37	228	0.167001845	ko04514
Porphyrin and chlorophyll metabolism	16	88	0.16742448	ko00860
Isoquinoline alkaloid biosynthesis	5	20	0.167619418	ko00950
Gastric acid secretion	26	155	0.175686908	ko04971
Synthesis and degradation of ketone bodies	4	15	0.181324296	ko00072
Carbon fixation pathways in prokaryotes	9	45	0.185225814	ko00720
Cardiac muscle contraction	18	103	0.185730548	ko04260
Legionellosis	29	177	0.188532669	ko05134
Circadian entrainment	29	178	0.195768103	ko04713
Cysteine and methionine metabolism	15	85	0.203329118	ko00270
Hematopoietic cell lineage	18	105	0.204909657	ko04640
Caprolactam degradation	4	16	0.206777957	ko00930
Degradation of aromatic compounds	6	28	0.209990636	ko01220
Cytokine-cytokine receptor interaction	38	244	0.224547389	ko04060
Adipocytokine signaling pathway	22	134	0.224769362	ko04920
Sulfur relay system	3	11	0.226289028	ko04122
Glutamatergic synapse	29	183	0.233980106	ko04724
Lysine degradation	26	163	0.239424624	ko00310
Pentose phosphate pathway	13	75	0.2412797	ko00030
Geraniol degradation	2	6	0.243801512	ko00281
Cutin, suberine and wax biosynthesis	2	6	0.243801512	ko00073
Ether lipid metabolism	13	76	0.254145651	ko00565
Phenylalanine metabolism	8	43	0.25419065	ko00360
Platelet activation	50	333	0.256067994	ko04611

Inositol phosphate metabolism	27	172	0.257428497	ko00562
Alzheimer's disease	46	306	0.26403277	ko05010
Carbon metabolism	40	264	0.266124344	ko01200
Two-component system	7	38	0.281931933	ko02020
AMPK signaling pathway	47	316	0.283295764	ko04152
Ubiquinone and other terpenoid-quinone biosynthesis	4	19	0.28800956	ko00130
Renin-angiotensin system	5	26	0.304171866	ko04614
Phototransduction	6	33	0.314431362	ko04744
ECM-receptor interaction	29	193	0.319072589	ko04512
Influenza A	53	365	0.323935217	ko05164
Phenylalanine, tyrosine and tryptophan biosynthesis	3	14	0.327843841	ko00400
Herpes simplex infection	61	425	0.339114218	ko05168
Taurine and hypotaurine metabolism	4	21	0.34436274	ko00430
Primary bile acid biosynthesis	4	21	0.34436274	ko00120
Drug metabolism - cytochrome P450	15	97	0.349495912	ko00982
Circadian rhythm - fly	5	28	0.353247242	ko04711
Fatty acid biosynthesis	7	42	0.361580003	ko00061
Calcium signaling pathway	45	313	0.363004909	ko04020
Pentose and glucuronate interconversions	14	91	0.363474013	ko00040
Glycosaminoglycan biosynthesis - keratan sulfate	4	22	0.372685925	ko00533
Tropane, piperidine and pyridine alkaloid biosynthesis	2	9	0.381629754	ko00960
Amphetamine addiction	17	114	0.385567582	ko05031
Estrogen signaling pathway	31	215	0.388680064	ko04915
Thyroid hormone synthesis	19	129	0.393688326	ko04918
Riboflavin metabolism	3	16	0.395890441	ko00740

Regulation of autophagy	6	37	0.402801601	ko04140
Biosynthesis of unsaturated fatty acids	6	37	0.402801601	ko01040
Glutathione metabolism	14	94	0.404934569	ko00480
NF-kappa B signaling pathway	22	152	0.409268917	ko04064
Olfactory transduction	9	59	0.417332901	ko04740
Various types of N-glycan biosynthesis	10	67	0.431432765	ko00513
Fructose and mannose metabolism	13	89	0.436256355	ko00051
Amyotrophic lateral sclerosis (ALS)	13	90	0.450707563	ko05014
Chemokine signaling pathway	45	325	0.453363753	ko04062
Pertussis	32	230	0.45875725	ko05133
Benzoate degradation	3	18	0.461945177	ko00362
Circadian rhythm	10	69	0.464622778	ko04710
2-Oxocarboxylic acid metabolism	6	40	0.468736967	ko01210
Serotonergic synapse	23	165	0.471041766	ko04726
Cholinergic synapse	31	225	0.480407024	ko04725
Proteoglycans in cancer	65	478	0.489279236	ko05205
Methane metabolism	12	86	0.499856093	ko00680
Melanogenesis	29	215	0.525553047	ko04916
Inflammatory mediator regulation of TRP channels	31	230	0.52601665	ko04750
Focal adhesion	65	484	0.527031228	ko04510
ABC transporters	14	103	0.529002205	ko02010
Nicotinate and nicotinamide metabolism	10	73	0.529748449	ko00760
Zeatin biosynthesis	1	5	0.531301245	ko00908
Drug metabolism - other enzymes	13	96	0.536070581	ko00983
Plant-pathogen interaction	4	28	0.536198077	ko04626

Prion diseases	8	59	0.548474227	ko05020
Thyroid cancer	10	75	0.561300793	ko05216
Fatty acid elongation	5	37	0.567587534	ko00062
Purine metabolism	44	334	0.577618623	ko00230
Retrograde endocannabinoid signaling	19	146	0.591161194	ko04723
Cocaine addiction	10	77	0.591959666	ko05030
Proximal tubule bicarbonate reclamation	7	54	0.596426548	ko04964
Vascular smooth muscle contraction	31	239	0.60539144	ko04270
Lysosome	29	224	0.607524318	ko04142
Sphingolipid metabolism	11	86	0.610248876	ko00600
Phosphonate and phosphinate metabolism	2	15	0.615830642	ko00440
Bladder cancer	13	102	0.616675928	ko05219
Salivary secretion	21	164	0.619395504	ko04970
Toxoplasmosis	35	272	0.625723777	ko05145
Starch and sucrose metabolism	16	126	0.625730091	ko00500
Glycosaminoglycan degradation	4	32	0.63234341	ko00531
Gap junction	22	174	0.64205903	ko04540
Pyrimidine metabolism	24	190	0.64706352	ko00240
Sulfur metabolism	2	16	0.647558025	ko00920
Aflatoxin biosynthesis	2	16	0.647558025	ko00254
Galactose metabolism	8	66	0.663731303	ko00052
N-Glycan biosynthesis	10	82	0.663858183	ko00510
p53 signaling pathway	15	122	0.668953928	ko04115
HTLV-I infection	69	539	0.672242233	ko05166

Glycosaminoglycan biosynthesis - heparan sulfate / heparin	4	34	0.675284854	ko00534
Basal cell carcinoma	11	91	0.677353833	ko05217
TNF signaling pathway	26	210	0.686171701	ko04668
SNARE interactions in vesicular transport	5	43	0.68797456	ko04130
Natural killer cell mediated cytotoxicity	22	179	0.688717686	ko04650
GnRH signaling pathway	24	195	0.691556236	ko04912
Dopaminergic synapse	28	227	0.697376155	ko04728
ErbB signaling pathway	23	188	0.698835875	ko04012
Wnt signaling pathway	35	282	0.700596061	ko04310
Dilated cardiomyopathy	20	165	0.704547363	ko05414
Folate biosynthesis	2	18	0.704710726	ko00790
Glycosphingolipid biosynthesis - ganglio series	2	18	0.704710726	ko00604
Taste transduction	5	44	0.705640882	ko04742
Non-homologous end-joining	3	27	0.707764049	ko03450
Collecting duct acid secretion	3	27	0.707764049	ko04966
Endocytosis	60	477	0.710028939	ko04144
Glycosylphosphatidylinositol(GPI)-anchor biosynthesis	4	36	0.714608166	ko00563
Dorso-ventral axis formation	6	53	0.715601094	ko04320
Oxytocin signaling pathway	42	339	0.717582509	ko04921
MAPK signaling pathway - yeast	7	62	0.725139266	ko04011
Amoebiasis	28	231	0.728021496	ko05146
Oocyte meiosis	24	200	0.732761322	ko04114
Glycerophospholipid metabolism	24	201	0.740582981	ko00564
VEGF signaling pathway	16	137	0.740869375	ko04370

Aldosterone-regulated sodium reabsorption	9	80	0.742860496	ko04960
Vitamin B6 metabolism	1	10	0.750766034	ko00750
Measles	25	211	0.755924395	ko05162
Progesterone-mediated oocyte maturation	23	197	0.770664036	ko04914
Propanoate metabolism	6	57	0.773093349	ko00640
Ras signaling pathway	48	396	0.77502564	ko04014
Malaria	7	66	0.777759723	ko05144
Rap1 signaling pathway	59	483	0.778916354	ko04015
Arrhythmogenic right ventricular cardiomyopathy (ARVC)	14	125	0.780243466	ko05412
Hedgehog signaling pathway	8	75	0.782812273	ko04340
Pathways in cancer	109	872	0.782847806	ko05200
T cell receptor signaling pathway	24	207	0.784464557	ko04660
TGF-beta signaling pathway	20	175	0.787263698	ko04350
Hypertrophic cardiomyopathy (HCM)	14	126	0.788999648	ko05410
Base excision repair	5	50	0.796792801	ko03410
Cytosolic DNA-sensing pathway	6	59	0.798365099	ko04623
Other glycan degradation	2	23	0.814369071	ko00511
Pantothenate and CoA biosynthesis	2	23	0.814369071	ko00770
Mineral absorption	7	70	0.822518123	ko04978
Adrenergic signaling in cardiomyocytes	34	295	0.827600987	ko04261
Cyanoamino acid metabolism	1	13	0.829383284	ko00460
Non-alcoholic fatty liver disease (NAFLD)	35	304	0.832214887	ko04932
Ribosome biogenesis in eukaryotes	13	123	0.83255894	ko03008
Huntington's disease	39	337	0.835874903	ko05016

Vasopressin-regulated water reabsorption	10	98	0.838406812	ko04962
Leukocyte transendothelial migration	31	273	0.839081761	ko04670
Phosphatidylinositol signaling system	25	225	0.84456185	ko04070
RIG-I-like receptor signaling pathway	11	108	0.848763423	ko04622
Styrene degradation	1	14	0.849631312	ko00643
Tight junction	38	335	0.862123316	ko04530
Pathogenic Escherichia coli infection	11	110	0.863141186	ko05130
Insulin signaling pathway	35	311	0.863858876	ko04910
Glioma	17	162	0.865392488	ko05214
Glycosphingolipid biosynthesis - globo series	1	15	0.867476743	ko00603
Colorectal cancer	18	171	0.868263236	ko05210
Jak-STAT signaling pathway	24	222	0.871197041	ko04630
Cell cycle - yeast	11	112	0.876410983	ko04111
NOD-like receptor signaling pathway	12	121	0.877682209	ko04621
MAPK signaling pathway	49	430	0.883110113	ko04010
Viral carcinogenesis	55	481	0.890695705	ko05203
Non-small cell lung cancer	14	141	0.891663211	ko05223
Glycosaminoglycan biosynthesis - chondroitin sulfate / dermatan sulfate	2	29	0.896924864	ko00532
Regulation of actin cytoskeleton	47	422	0.907625654	ko04810
Meiosis - yeast	9	100	0.911426739	ko04113
Alanine, aspartate and glutamate metabolism	5	62	0.911630402	ko00250
MicroRNAs in cancer	33	308	0.912479697	ko05206
Acute myeloid leukemia	12	128	0.914676114	ko05221
mRNA surveillance pathway	13	137	0.914793136	ko03015

RNA transport	26	250	0.915458252	ko03013
Endocrine and other factor-regulated calcium reabsorption	10	111	0.920165394	ko04961
Chagas disease (American trypanosomiasis)	29	277	0.920370719	ko05142
Protein processing in endoplasmic reticulum	36	336	0.920888786	ko04141
Long-term depression	9	102	0.921380626	ko04730
Synaptic vesicle cycle	8	93	0.923217499	ko04721
FoxO signaling pathway	35	329	0.924529988	ko04068
Ubiquitin mediated proteolysis	23	228	0.927067509	ko04120
Streptomycin biosynthesis	2	33	0.931347903	ko00521
Vibrio cholerae infection	10	114	0.932980767	ko05110
Bacterial invasion of epithelial cells	21	213	0.934079372	ko05100
Axon guidance	33	316	0.934201395	ko04360
Notch signaling pathway	12	133	0.934924004	ko04330
Homologous recombination	3	45	0.93508558	ko03440
Apoptosis	16	170	0.937562677	ko04210
Mismatch repair	2	34	0.938071286	ko03430
B cell receptor signaling pathway	13	145	0.944281291	ko04662
Endometrial cancer	11	127	0.945950949	ko05213
cGMP-PKG signaling pathway	37	357	0.949229107	ko04022
Alcoholism	14	157	0.952189282	ko05034
Melanoma	12	140	0.956253178	ko05218
Ribosome	24	249	0.957094514	ko03010
Fc gamma R-mediated phagocytosis	20	215	0.960092276	ko04666
Epithelial cell signaling in Helicobacter pylori infection	11	134	0.964497503	ko05120
Toll-like receptor signaling pathway	13	156	0.970189362	ko04620

African trypanosomiasis	2	41	0.970310056	ko05143
Basal transcription factors	4	65	0.970415137	ko03022
Long-term potentiation	11	137	0.970529433	ko04720
Sphingolipid signaling pathway	24	258	0.971573733	ko04071
Osteoclast differentiation	19	213	0.971951407	ko04380
Choline metabolism in cancer	21	232	0.973000141	ko05231
cAMP signaling pathway	43	426	0.973673335	ko04024
Prostate cancer	20	224	0.974479108	ko05215
Proteasome	3	55	0.974519126	ko03050
Transcriptional misregulation in cancer	30	314	0.9747739	ko05202
mTOR signaling pathway	10	130	0.975377916	ko04150
Small cell lung cancer	22	243	0.975442923	ko05222
RNA degradation	12	151	0.977458005	ko03018
Carbohydrate digestion and absorption	5	80	0.978676486	ko04973
Neurotrophin signaling pathway	22	247	0.979884491	ko04722
Hippo signaling pathway	26	286	0.982296328	ko04390
MAPK signaling pathway - fly	1	31	0.98244941	ko04013
Chronic myeloid leukemia	15	186	0.984328871	ko05220
Hepatitis B	29	316	0.984576481	ko05161
Prolactin signaling pathway	12	157	0.984586981	ko04917
Fanconi anemia pathway	5	86	0.987184726	ko03460
Salmonella infection	13	171	0.988147337	ko05132
Adherens junction	22	265	0.992205137	ko04520
Maturity onset diabetes of the young	1	38	0.992754397	ko04950
Cell cycle	21	263	0.99489858	ko04110

Signaling pathways regulating pluripotency of stem cells	21	267	0.995954855	ko04550
Pancreatic cancer	12	177	0.996007811	ko05212
Fc epsilon RI signaling pathway	6	116	0.997393931	ko04664
HIF-1 signaling pathway	19	256	0.997603726	ko04066
DNA replication	2	64	0.997639045	ko03030
Central carbon metabolism in cancer	10	163	0.997681439	ko05230
Hepatitis C	15	222	0.998461618	ko05160
Nucleotide excision repair	2	68	0.998498724	ko03420
Shigellosis	7	137	0.998817869	ko05131
Thyroid hormone signaling pathway	21	297	0.999363264	ko04919
Type II diabetes mellitus	4	106	0.999402866	ko04930
Epstein-Barr virus infection	29	382	0.999534534	ko05169
Hippo signaling pathway - fly	7	149	0.999590834	ko04391
Amino sugar and nucleotide sugar metabolism	2	86	0.999810099	ko00520
Renal cell carcinoma	8	172	0.99984154	ko05211
Spliceosome	10	200	0.999884654	ko03040
