# Supplementary figures and images for: Transcriptomic Analysis of Liver Tissue of Black Sea Bass (Centropristis striata) Exposed to High Nitrogen Environment
Source: Genes (Basel). 2023 Jul 13;14(7):1440. doi: 10.3390/genes14071440 (PMC10378819; doi:10.3390/genes14071440)

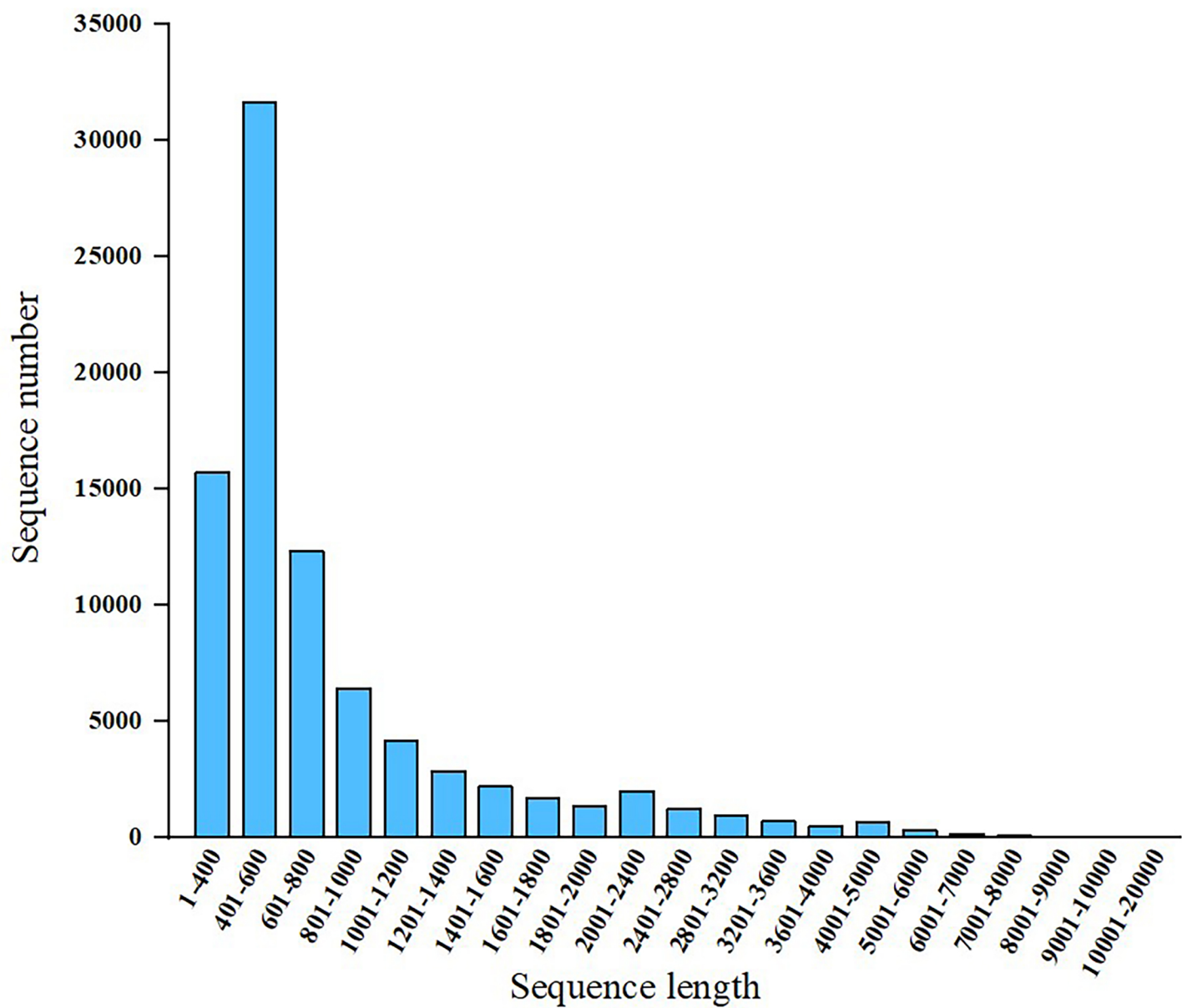

Supplement: Supplementary file 1 [file genes-14-01440-s001.zip › Figure S1.pdf]

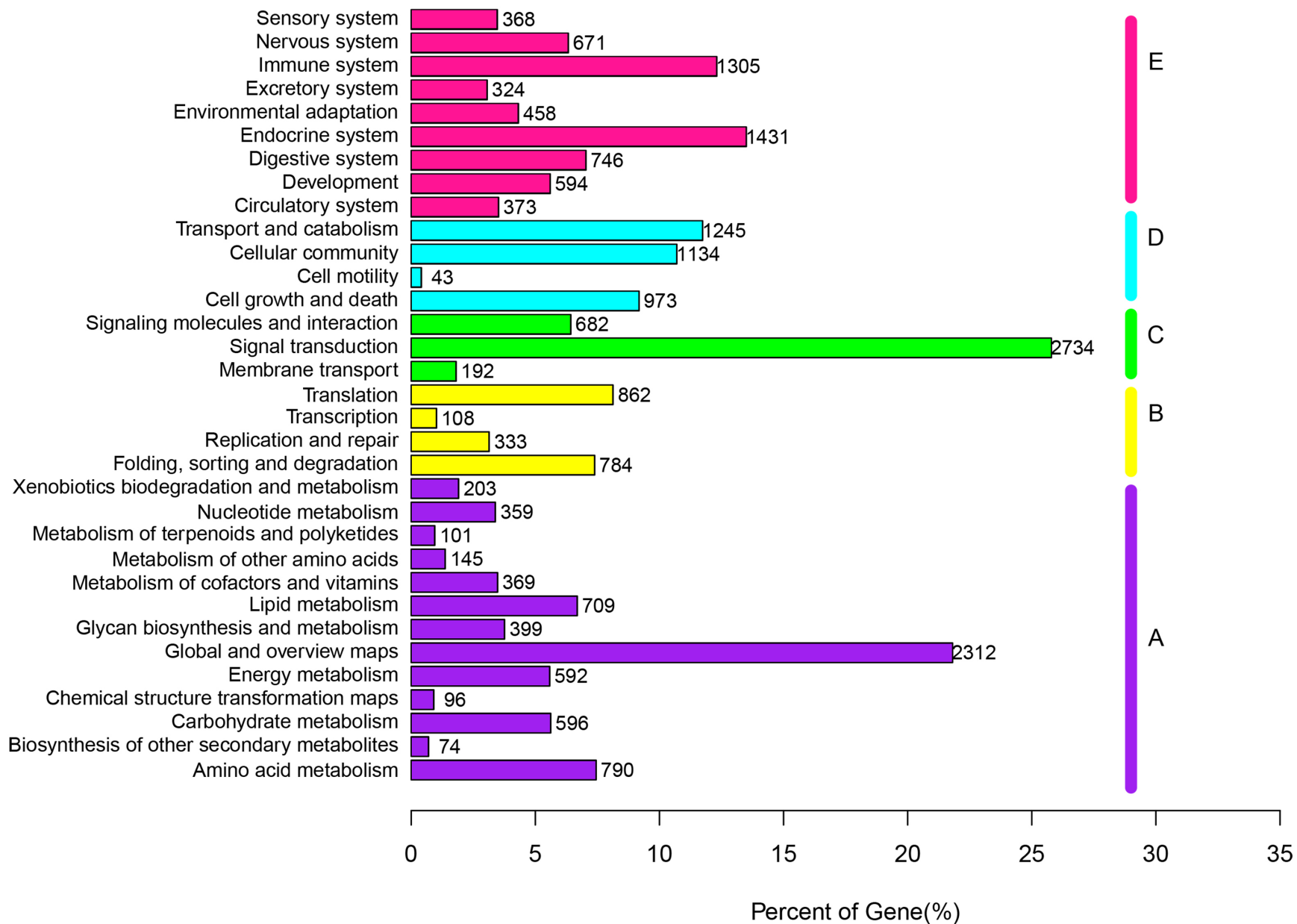

Supplement: Supplementary file 1 [file genes-14-01440-s001.zip › Figure S3.pdf]

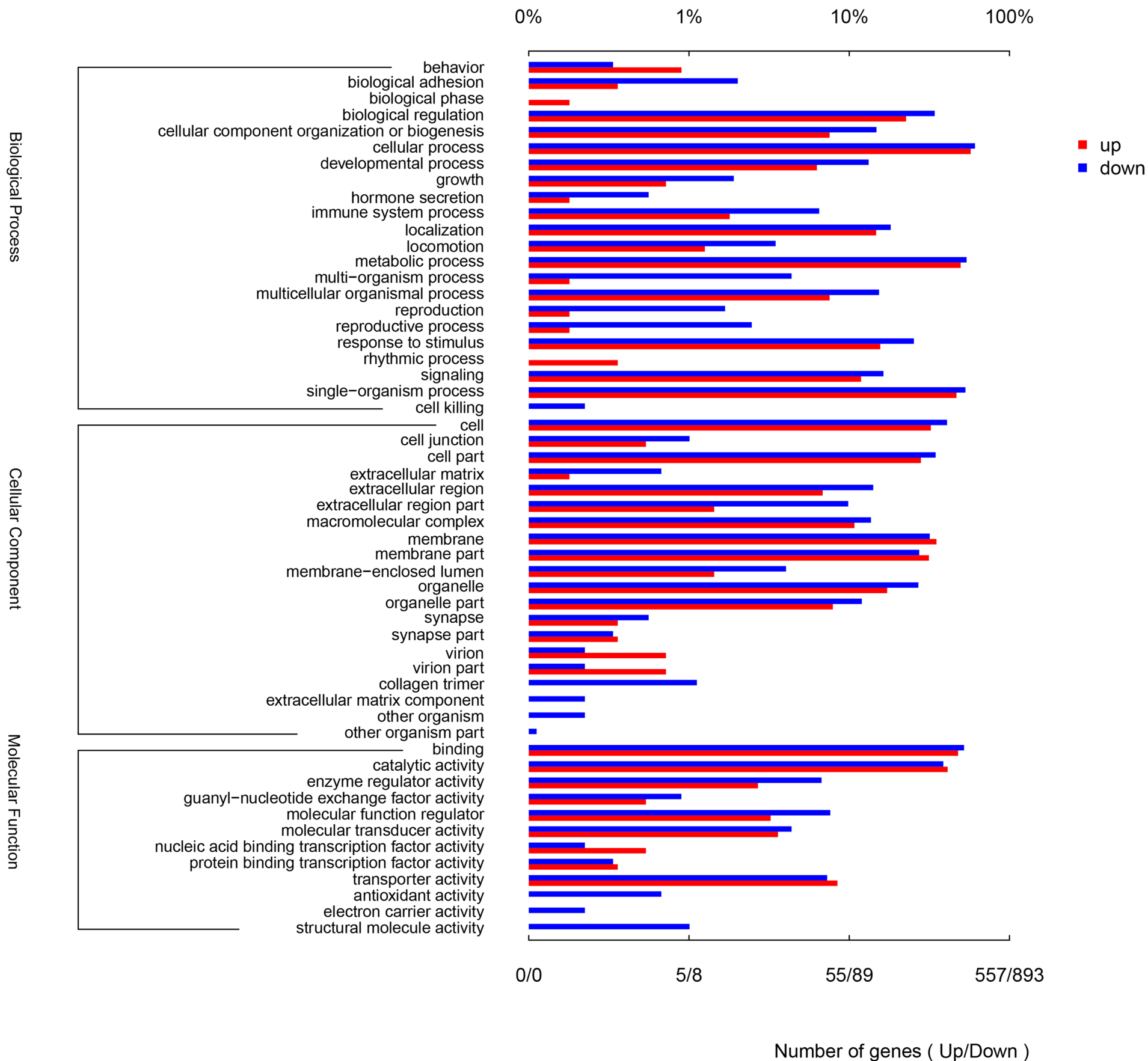

Supplement: Supplementary file 1 [file genes-14-01440-s001.zip › Figure S4.pdf]

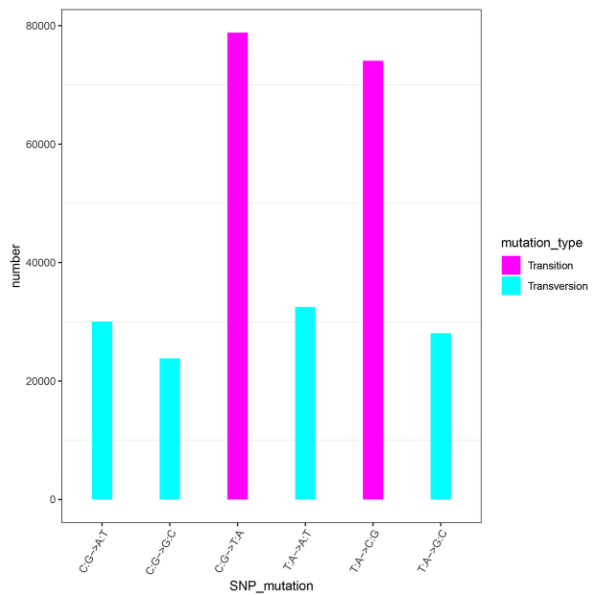

**ZD-1**

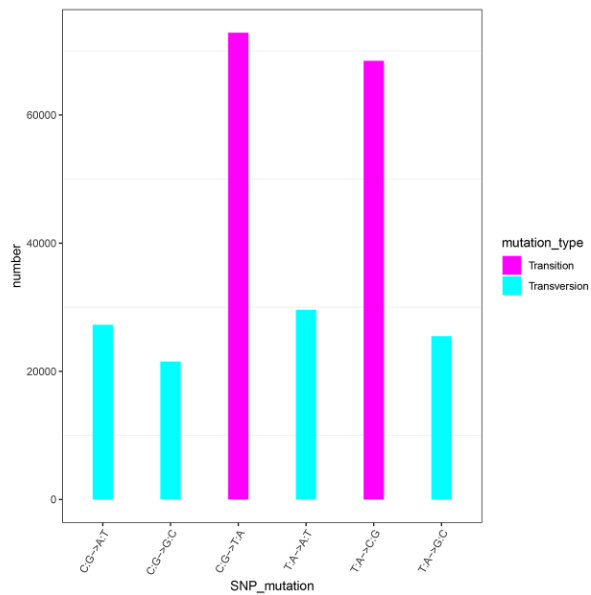

**ZD-2**

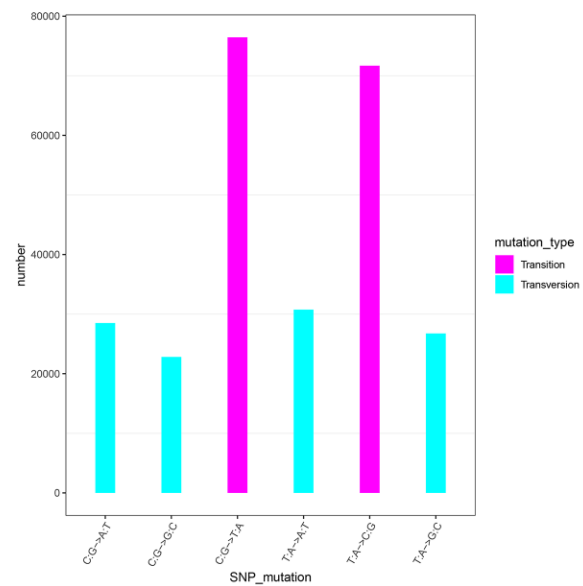

**ZD-3**

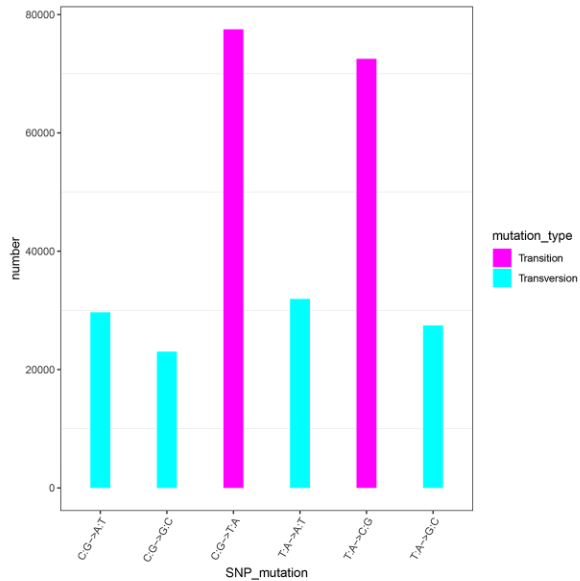

**ZS-1**

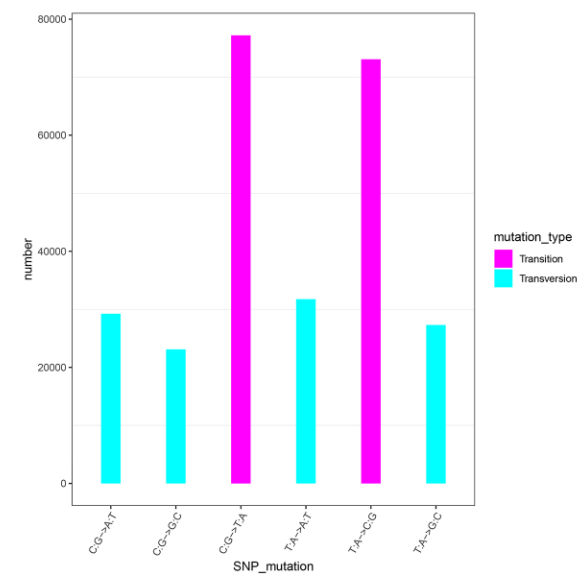

**ZS-2**

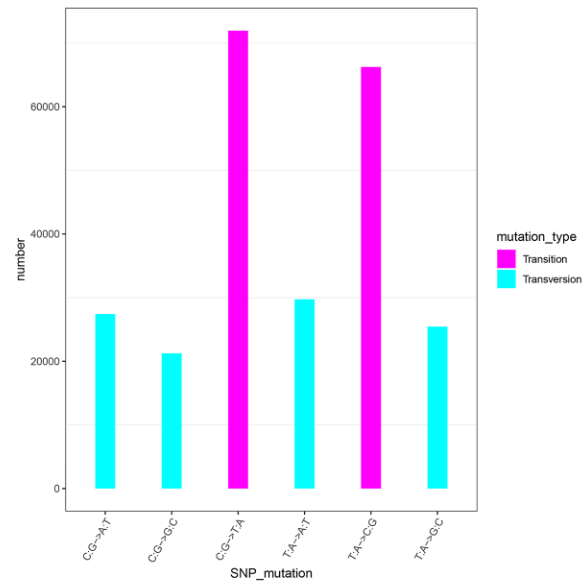

**ZS-3**

Supplement: Supplementary file 1 [file genes-14-01440-s001.zip › Figure S5.pdf]

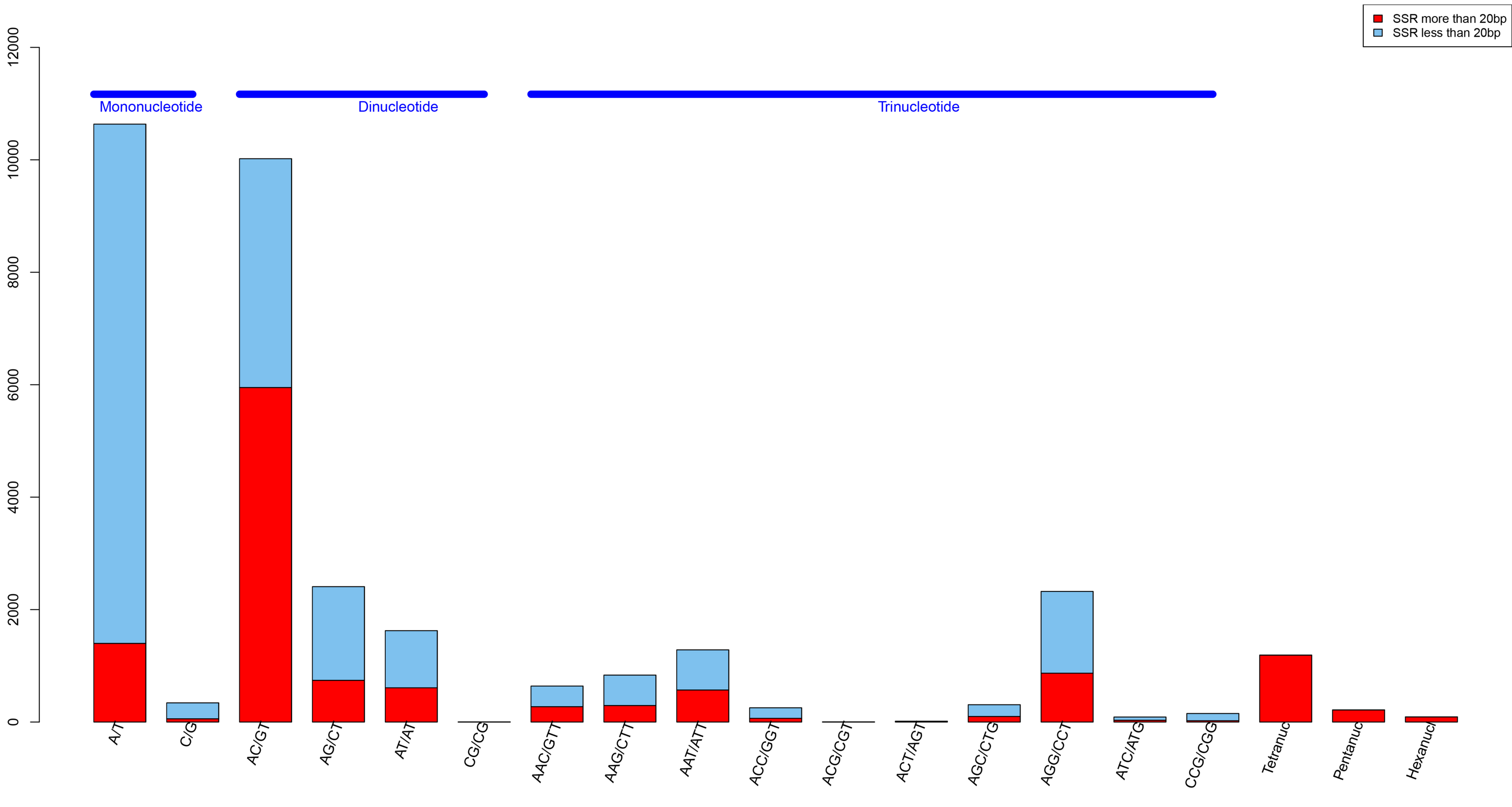

Supplement: Supplementary file 1 [file genes-14-01440-s001.zip › Figure S6.pdf]
